# Genomes of nine biofilm-forming filamentous strains of Cyanobacteria (genera *Jaaginema, Scytonema*, and *Karukerafilum* gen. nov.) isolated from mangrove habitats of Guadeloupe (Lesser Antilles)

**DOI:** 10.1093/femsmc/xtad024

**Published:** 2023-12-14

**Authors:** Sébastien Halary, Charlotte Duval, Benjamin Marie, Cécile Bernard, Bérénice Piquet, Olivier Gros, Marie-Lise Bourguet-Kondracki, Sébastien Duperron

**Affiliations:** Molécules de Communication et Adaptation des Microorganismes, UMR 7245 CNRS, Muséum National d'Histoire Naturelle, 75005 Paris, France; Molécules de Communication et Adaptation des Microorganismes, UMR 7245 CNRS, Muséum National d'Histoire Naturelle, 75005 Paris, France; Molécules de Communication et Adaptation des Microorganismes, UMR 7245 CNRS, Muséum National d'Histoire Naturelle, 75005 Paris, France; Molécules de Communication et Adaptation des Microorganismes, UMR 7245 CNRS, Muséum National d'Histoire Naturelle, 75005 Paris, France; Molécules de Communication et Adaptation des Microorganismes, UMR 7245 CNRS, Muséum National d'Histoire Naturelle, 75005 Paris, France; Institut de Systématique, Evolution, Biodiversité (ISYEB), Muséum National d'Histoire Naturelle, CNRS, Sorbonne Université, EPHE, Université des Antilles, 97110 Pointe-à-Pitre, France; Molécules de Communication et Adaptation des Microorganismes, UMR 7245 CNRS, Muséum National d'Histoire Naturelle, 75005 Paris, France; Molécules de Communication et Adaptation des Microorganismes, UMR 7245 CNRS, Muséum National d'Histoire Naturelle, 75005 Paris, France

**Keywords:** genomes, biofilms, filamentous, strains, Cyanobacteria, mangrove

## Abstract

Biofilm-forming cyanobacteria are abundant in mangrove ecosystems, colonizing various niches including sediment surface and periphyton where they can cover large areas, yet have received limited attention. Several filamentous isolates were recently isolated from Guadeloupe, illustrating the diversity and novelty present in these biofilms. In this study, nine strains belonging to three novel lineages found abundantly in Guadeloupe biofilms are characterized by genome sequencing, morphological and ultrastructural examination, metabolome fingerprinting and searched for secondary metabolites biosynthesis pathways. Assignation of two lineages to known genera is confirmed, namely *Scytonema* and *Jaaginema*. The third lineage corresponds to a new Coleofasciculales genus herein described as *Karukerafilum* gen. nov. The four strains belonging to this genus group into two subclades, one of which displays genes necessary for nitrogen fixation as well as the complete pathway for geosmin production. This study gives new insights into the diversity of mangrove biofilm-forming cyanobacteria, including genome-based description of a new genus and the first genome sequence available for the genus *Jaaginema*.

## Teaser

Genome of nine filamentous cyanobacterial strains from mangrove habitats of Guadeloupe.

## Introduction

Biofilm-forming cyanobacteria (phylum Cyanobacteriota) can contribute significantly to ecosystems primary production, in particular in the tropics where they can cover very large areas of sediment, rocks or a variety of alive or dead biological surfaces. They are mainly filamentous in biofilms, and can either colonize freshwater or shallow-water marine environments such as mangrove or rivers (Guidi-Rontani et al. 2014, Alvarenga et al. 2015, Shah et al. 2017). These biofilms also harbor many other prokaryotic and eukaryotic lineages that interact closely, competing or cooperating in nutrient cycling, and production of protective compounds (Rigonato et al. 2013, Basak et al. 2016, Allard et al. 2020). Aside from carbon fixation, cyanobacteria in biofilms can contribute to nutrient cycling through nitrogen fixation, accumulation of calcium, magnesium and phosphorous (Lovelock et al. 2010). Interestingly, grazing does not seem to cause massive damage on biofilms, suggesting the existence of defense molecules to which cyanobacteria, as producers of various bioactive compounds, may contribute actively (Demay et al. 2019). Indeed, these phototrophs are known to produce a wide diversity of bioactive compounds (Demay et al. 2019). These range from cyanotoxins that are of major significance to ecosystem, animal and human health, to molecules of pharmacological interest such as Brentuxymab vedotin, based on dolastatin 10 from *Symploca*, which reached the market for the treatment of Hodgkin's lymphoma (Mi et al. 2017, Shah et al. 2017, Demay et al. 2019).

Aquatic cyanobacteria from tropical areas are regarded as a relatively accessible yet untapped source of new taxa and biomolecules (Alivisatos et al. 2015, Allard et al. 2020), and increasing efforts are being deployed to characterize their diversity. Currently, 184 reference genomes are available from cyanobacteria species, a subset of the 5700 described cyanobacterial species, itself certainly a small subset of the group's true diversity, which remains vastly underestimated (estimates up to 8000 species have been proposed) (Nabout et al. 2013, Komarek et al. 2014, Strunecký et al. 2023). The tropical regions and the marine benthic compartment are particularly under-explored compared to the potential diversity their harbor (Alvarenga et al. 2015). In a recent study, a high diversity of novel benthic biofilm-forming cyanobacterial lineages was evidenced in coastal habitats of Guadeloupe (Lesser Antilles) using strain isolation and 16S rRNA comparative gene sequence analysis (Duperron et al. 2020). Among these, several potential species or genera were represented by multiple distinct isolates. Further exploring the diversity (e.g. taxonomical, inter- or intraspecific), characteristics and potential of these strains requires whole length genome sequencing, morphological, ultrastructural and metabolomic analysis. Concerning genome sequencing, more and more studies point towards high heterogeneity in genome contents among closely related strains, emphasizing the need to sequence beyond individual strains genomes, but rather the genomes of multiple closely related strains to get a glimpse into the so-called pangenome of a species-level taxon (Meyer et al. 2017, Pérez-Carrascal et al. 2019, Willis and Woodhouse 2020). Evaluating this micro-scale diversity is key to understanding the actual breadth of a species ecological niche (Dvořák et al. 2023, Halary et al. 2023).

In this study, three novel lineages of filamentous cyanobacteria isolated from coastal habitats in Guadeloupe were explored by sequencing the genomes of two to four strains per lineage and by studying their morphology, ultrastructure and metabolomic profiles. These lineages were provisionally assigned to genera *Jaaginema, Oscillatoria* and *Scytonema* and were selected because they represent a significant fraction of the bacteria occurring in the biofilms from which they were isolated (Duperron et al. 2020), several strains are available, and because they represent lineages for which little genomic information is currently available in the literature. Genome of each strain is sequenced, and a comparative analysis using a polyphasic approach is conducted to ascertain their taxonomic affiliation, identify their metabolic capabilities, and document inter-strain variability, with a focus on secondary metabolites biosynthesis pathways. This study provides the first genomes from Guadeloupe strains, and the first *Jaaginema* genomes. The strains previously assigned to *Oscillatoria* appear to belong to a new genus, with *Karukerafilum mangrovensis* herein described as representative species. Intra-clade genomic variability reveals major differences between closely related strains that may explain the intra- and inter-taxa diversity and the ecological success of these cyanobacteria.

## Material and methods

### Origin of strains

The nine strains analyzed in this study were isolated from three distinct locations in Guadeloupe (Duperron et al. 2020). Dense green-to-brown benthic or periphytic biofilms were sampled in July 2018 in the station of Manche-à-Eau lagoon close to *Rhizophora mangle* roots, and epiphytic biofilms were collected from two stations located alongside the Canal des Rotours, a 6 km-long canal connecting the marine lagoon to the city of Morne-à-l'eau through various mangrove (Supplementary Fig. 1). In accordance with Article 17, paragraph 2, of the Nagoya Protocol on Access and Benefit-sharing, a sampling permit was issued and published (https://absch.cbd.int/en/database/CNA/ABSCH-CNA-FR-240495). Back to the laboratory, a fraction of each biofilm was frozen for community characterization (see below). Another fraction was examined under a binocular and individual cyanobacterial morphotypes were isolated manually to plates containing solid Z8 medium (Rippka 1988) containing 0, 20, and 35 g.L^−1^ salt (Instant Ocean, Aquarium Systems, France). Cultures were stabilized and maintained in the Paris Museum Collection (PMC) (Duperron et al. 2020). Among the recovered culturable strains (described in Duperron et al. 2020), nine were selected for subsequent genome sequencing. For simplification purposes, these strains will be referred in the rest of the manuscript as PMC 1050, PMC 1051, PMC 1068, PMC 1076, PMC 1069, PMC 1070, PMC 1078, PMC 1079, and PMC 1080 (strains origin are summarized in Table 1).

**Table 1.**
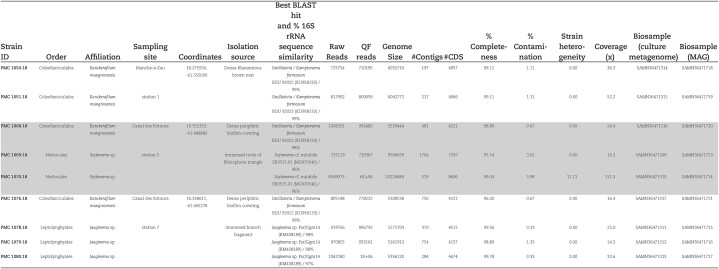
Strains isolated from Guadeloupe habitats and associated genome characteristics. Strain ID corresponds to the reference number in the Paris Museum Collection (PMC) of cyanobacteria from which strains are available upon request. Affiliation is according to the 16SrRNA-based phylogenetic analysis displayed on Fig. S2 and to the distance matrix in Table S1.

### Metabarcoding and biofilm composition

DNA was extracted from the 3 biofilms using the ZymoBIOMICS Fecal/Soil Kit (Zymo Research, CA) according to the manufacturer's instructions, including a 3 min disruption of cells using ceramic beads. PCR using universal primers to amplify the V4-V5 region of the 16S rRNA-encoding gene was performed as described in (Duperron et al. 2020), using primers 515F and 926 R (Parada et al. 2016, Newman and Cragg 2017) and sequenced on an Illumina MiSeq platform (2×300 bp, paired-end sequencing, Genoscreen, France). Company-provided mock communities of known composition were used as an internal control for the whole sequencing process. Raw reads were deposited into the GENBANK Sequence Read Archive (SRA, Bioproject PRJNA994497, Biosamples SAMN36430094-6).

Sequence analysis was performed using QIIME2 (Caporaso et al. 2012, Callahan et al. 2017). Amplicon Sequence Variants (ASVs) were identified using DEBLUR (Amir et al. 2017) using default parameters, *i.e*. a maximal probability for indels of 0.01 and mean read error rate of 0.5% for normalization. Chimeric sequences were identified and discarded using UCHIME (*de novo* chimera detection) (Edgar et al. 2011), and the taxonomic affiliations were obtained by using the sklearn-based classifier (GreenGenes 13–8-99 release). Sequences matching “Eukaryota”, “Chloroplast” and “Mitochondria” were discarded. AVSs corresponding or highly similar to the nine strains sequenced in this study were searched for, and their abundance among total reads was computed.

### Cultivation and strains genome sequencing

Biomass was produced from the nine selected isolates for two months in increasing volumes of liquid Z8 or Z8X media (25 ± 1°C; 15 µmol.m^−2^.s^−1^ white light; 16 h light: 8 h dark) with 20 g.L^−1^ Instant Ocean salt (Aquarium Systems, France) (Rippka 1988). DNA was then extracted from culture-derived biomass using the ZymoBIOMICS Fecal/Soil Kit (Zymo Research, CA) following manufacturer's instructions including a 3 min disruption of cells using ceramic beads. Concentrations were measured using Nanodrop and Qubit (Thermo Fisher). DNA sequencing was performed using an Illumina MiSeq platform on 2×250 bp reads libraries (Genoscreen, France). Assemblies were achieved using SPAdes v3.15.4 (with ‘–meta’ option and default parameters) (Bankevich et al. 2012) and the resulting scaffolds were then taxonomically annotated using CAT (with default parameters) (von Meijenfeldt et al. 2019). All scaffolds affiliated to Cyanobacteria constituted the present genomes, for which completeness and contamination were assessed using CheckM (Parks et al. 2015).

### Gene annotation and comparative genome analysis

Coding DNA sequences were predicted using Prodigal (Hyatt et al. 2010) and functionally annotated using eggnog-mapper v2 (Huerta-Cepas et al. 2019). Clusters of orthologous genes among strains were determined with Orthofinder (Emms and Kelly 2019). Secondary metabolite biosynthesis gene clusters were identified using antiSMASH (Blin et al. 2021). Metagenome sequences from the cultures, and Metagenome-Assembled-Genomes (MAGs) were deposited in the SRA database (Bioprojet: PRJNA994497, Table 1).

### Phylogenomic inference

All available reference genomes of Cyanobacteria with a complete assembly level in December 2021 were downloaded from NCBI (173 genomes in total), as well as a set of 3 genomes for an outgroup constitution, namely *Anthocerotibacter panamensis* C109, *Gloeobacter kilaueensis* JS1 and *Gloeobacter violaceus* PCC 7421. FetchMG was then used to retrieved single copy marker genes (MG) present in this dataset and our 9 cyanobacterial strain genomes (Kultima et al. 2012). In order to maximize the number of marker genes, genomes displaying a low number of MG were discarded. In total, 144 genomes sharing 30 MG altogether were kept for the phylogenomic analysis. First, a multiple alignment was achieved for each MG using MAFFT with local alignment iterative refinement option (Katoh et al. 2019). All alignments were then concatenated and refined using BMGE with default options (Criscuolo and Gribaldo 2010). Finally, the resulting alignment of length 6457 aa was used to build a phylogenomic tree using RaxML v8.2.12 (WAGGAMMA model, 100 bootstraps) (Stamatakis et al. 2012).

### Metabolomic profiling

For each cyanobacterial strain, cellular biomasses were obtained from 500-mL cultures in 2-L Erlenmeyer flasks with a photon flux density of 6 µmol.m^−2^.s^−1^ and a 13:11 h light: dark cycle, with the aim to produce enough biomass in standardized conditions for the different strains. Cyanobacterial cells were centrifuged at 4,000 rpm for 10 min. The supernatants were discarded and the pellets were freeze-dried and lyophilized (Freezone 2.5 L, Labconco, Kansas City, USA). Then, the lyophilized cells were weighted then sonicated 2 min in 80% methanol with a constant ratio of 100 µL of solvent for 1 mg of dried biomass and centrifuged at 4°C (12,000 g; 5 min). Two microliters of the supernatant were analysed in triplicate with an ultra-high-performance liquid chromatograph (UHPLC Ultimate 3000, Thermo, Waltham, MA, USA) using a Polar Advances II 2.5 pore C18 column (Thermo, Waltham, MA, USA) at a 300 µL·min^−1^ flow rate with a linear gradient of acetonitrile in 0.1% formic acid (5%–90% of 21 min) coupled with a high-resolution mass spectrometer. The eluted metabolite contents were analysed using an electrospray ionization hybrid quadrupole time-of-flight (ESI-QqTOF) high resolution mass spectrometer (Maxis II ETD, Bruker). Positive and negative autoMSMS mode was used with information dependent acquisition (IDA), on the 50-1500 *m/z* range at 2 Hz or between 2-8 Hz speed, for MS and MS/MS respectively, according to the relative intensity of the parent ions, in consecutive cycle times of 2.5 s, with active exclusion of previously analysed parents. The data were analysed with the MetaboScape 4.0 software (Bruker) in order to automatically perform internal recalibration (<0.5 ppm), search and group all together classical adduct forms (M+H^+^, M+2H^+^, M+3H^+^, M+Na^+^, M+K^+^ and M+NH_4_+) using a threshold value of 0.8 value for the co-elution coefficient factor. Metabolite annotation was attempted according to the precise mass of the molecules and their respective MS/MS fragmentation patterns with regards to MS/MS libraries (NIH, GNPS, EMBL, MassBank and ReSpect) and the CyanoMet database (Jones et al. 2020, 2021) of over 2 100 cyanobacterial metabolites and confirmed with 36 commercially available standard molecules from the following cyanobacterial metabolite families (e.g. cyanopeptolins, aeruginosins, microginins, anabaenopeptins, aerucyclamides, microcystins, saxitoxins, anatoxins, and cylindrospermopsins) that were analyzed similarly in mass spectrometer (Maxis II ETD, Bruker) (Olivon et al. 2018, Kim Tiam et al. 2019).

The molecular network was created using the MetGem v1.2.2 software (Olivon et al. 2018) from the whole MS/MS data (in mgf format) for the two extracted out of the nine cyanobacteria. The network was created where edges were filtered to have a cosine score above 0.65 and more than four matched peaks. Further edges between two nodes were kept in the network only if each of the nodes appeared in each other's respective top 10 most similar nodes. The MetGem database search function was used to screen each spectrum with GNPS spectral libraries.

### Light and electron microscopy analyses

Light microscopy photographs of the specimens were taken with an AxioCam MRc digital camera coupled to an Axio ImagerM2 Zeiss microscope. Cell and filament width, length, morphology, color and motility were determined. Strains were identified morphologically using the updated taxonomic literature (Komarek et al. 2014, Komárek and Johansen 2015). For electron microscopy, strains from a growing culture were fixed with 2.5% glutaraldehyde, 2% paraformaldehyde, 0.18 M sucrose and 0.1% picric acid in 0.1 M Sorensen phosphate buffer (pH 7.4) for 1 hour at RT. Cells were post-fixed with 1% osmium tetroxide during 1 hour in the same buffer (A hour, RT), then rinsed with distilled water and dehydrated in a graded ethanol series (30%, 50%, 70, 85%, 95% and 100%, 15 min each). Cyanobacteria were then embedded in Epon resin in an increasing gradient of resin in ethanol. Samples were sectioned (60 nm, thick) with an ultramicrotome (RMC Ultramicrotome PowerTome XL) and transferred onto 150 mesh copper formvar grids. Grids were stained with uranyl acetate saturated in 50% ethanol and examined under a transmission electron microscope (Hitachi 7700, Japan) under an acceleration voltage of 80 kV.

## Results

### Genome sequencing and abundance within sampled biofilms

**Figure 1.**
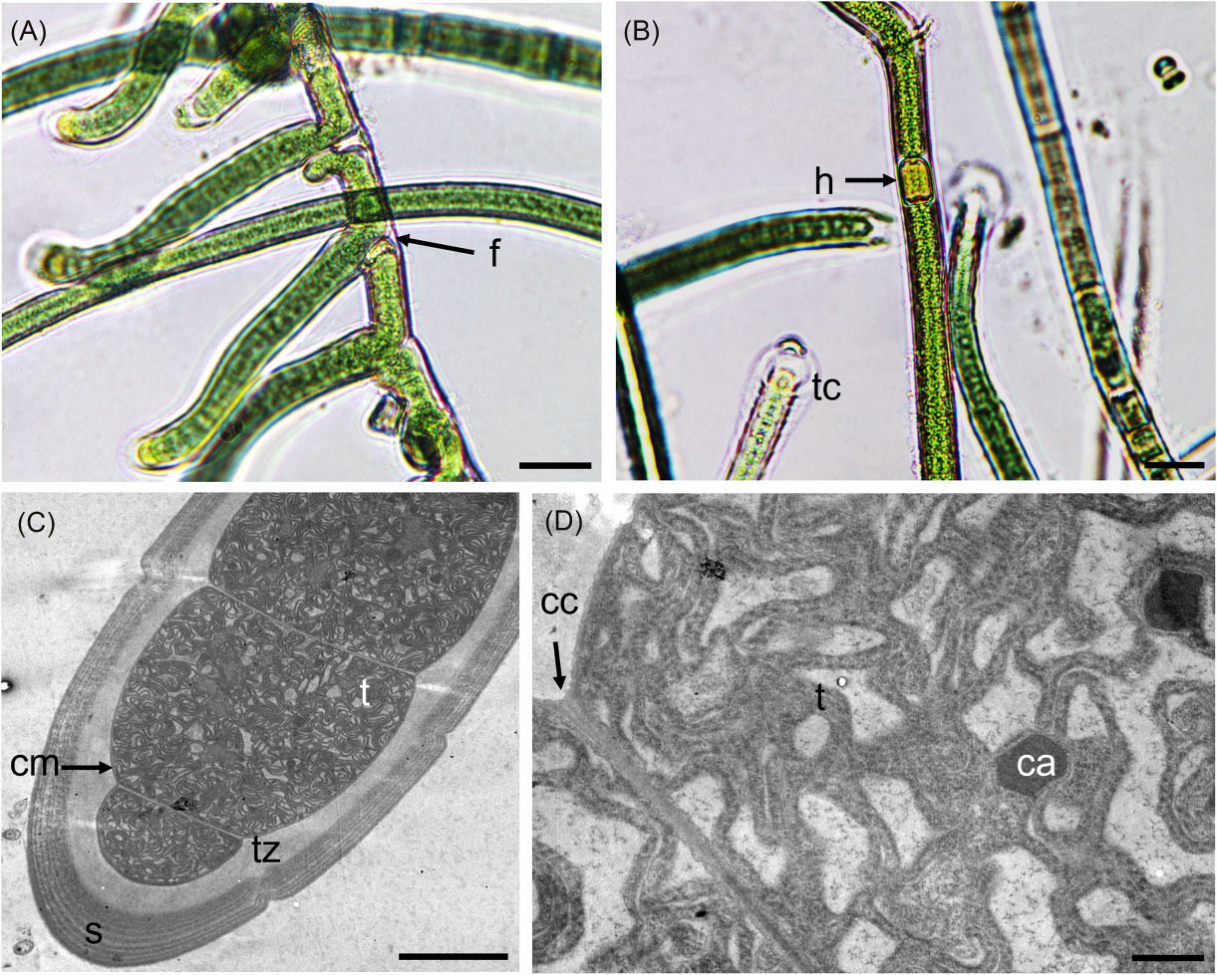
Light microscopy (A and B) and TEM (C and D) micrographs of *Scytonema* sp. PMC 1069.18 (Order Nostocales) from Guadeloupe mangroves. Abbreviations: cc: cross-wall constriction, ca: carboxysome, cm: cytoplasmic membrane, f: false branching, h: heterocyte, s: sheath, t: thylakoids, tc: terminal cell and tz: transparent zone. Scale bars A and B = 20 µm, C = 5 µm and D = 500 nm.

**Figure 2.**
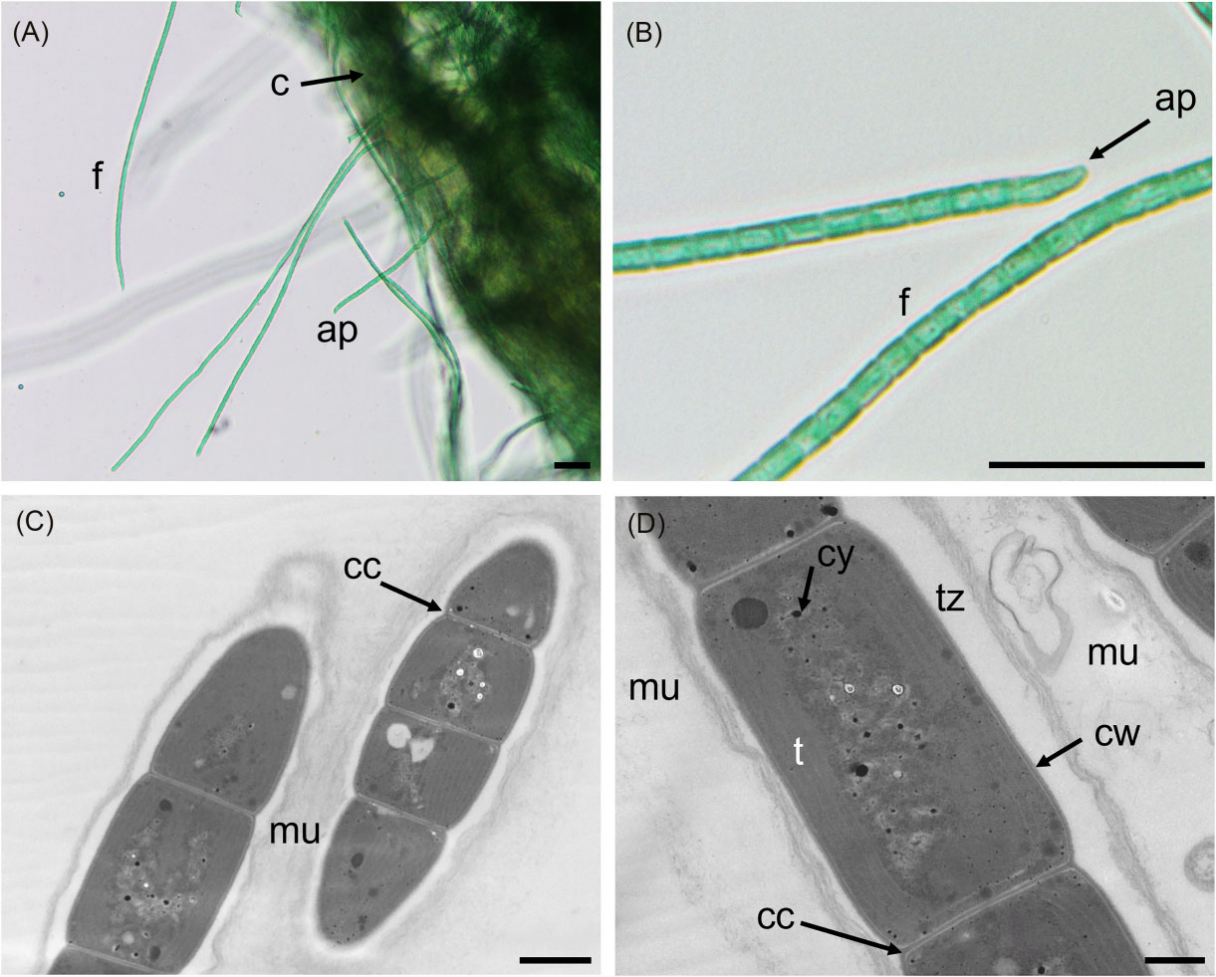
Light microscopy (A and B) and TEM (C and D) micrographs of *Jaaginema* sp. PMC 1079.18 (Order Leptolyngbyales) from Guadeloupe mangroves. Abbreviations: ac: apical cell, c: colony, cc: constriction, cw: cell wall, cy: cyanophycin granules, f: filament, mu: mucilage and t: thylakoids. Scale bars A and B = 20 µm, C = 1 µm and D = 500 nm.

**Figure 3.**
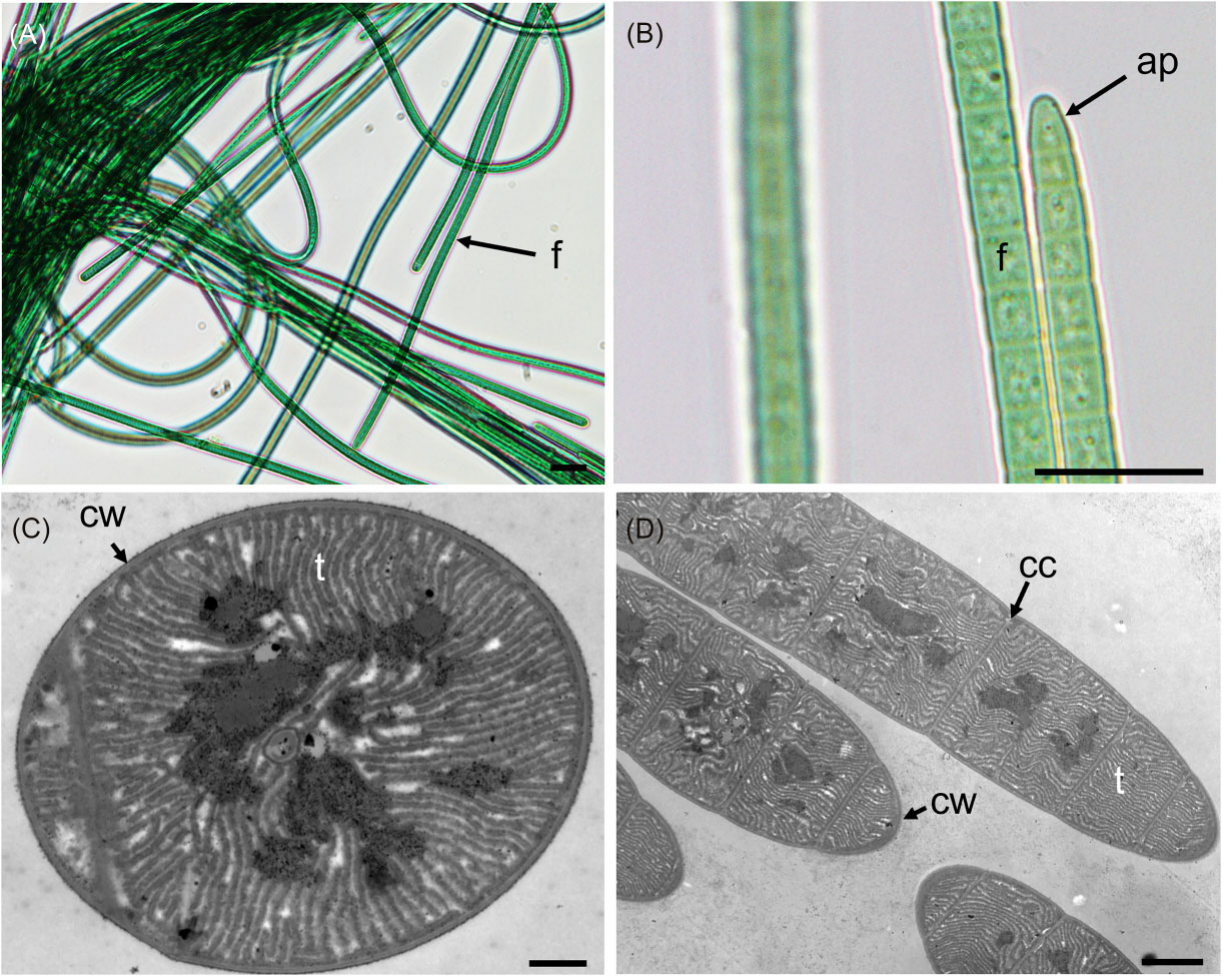
Light microscopy (A and B) and TEM (C and D) micrographs of *Karukerafilum mangrovensis* PMC 1051.18 (Order Coleofasciculales) from Guadeloupe mangroves. Abbreviations: cc : cell constriction, cy: cyanophycin granules, cw : cell wall, f: filament and t: thylakoids. Scale bars A = 20 µm, B = 2 µm, C and D = 500 nm.

Genome sequences were obtained for all nine selected filamentous strains. Based on re-analysis of 16S rRNA gene sequencing (Supplementary Fig. 2), two of these strains were provisionally assigned to genus *Scytonema* (Order Nostocales; PMC 1069; 1070, Fig. 1), three to genus *Jaaginema* (formerly Order Synechococcales, recently reclassified in the Leptolyngbyales (Strunecký et al. 2023): PMC 1078, 1079; 1080; Fig. 2), and four to Order Coleofasciculales (although a previous study initially suggested genus *Oscillatoria*); PMC 1050, 1051, 1068, 1076, Fig. 3) (Duperron et al. 2020). Genome sizes were between 5.16 and 10.2Mb (Table 1). Completeness values were high, above 98.6% except for strain PMC 1069 (95.5%) and 1076 (96.0%), and coverage ranged from 14.1 to 111.3x. Contamination was low, except for the two *Scytonema*-affiliated strains PMC 1069 and 1070 which displayed 3.05% and 4.0% contamination, respectively. Strain heterogeneity was high in strain PMC 1070 (11.1).

Amplicon Sequence Variants identical to 16S rRNA sequences of O/Coleofasciculales and O/Leptolyngbyales strains from this study represented 2% and 5.7% of the reads in the biofilm from station 7 where PMC strains were isolated, respectively. O/Nostocales strains represented 50% of 16S rRNA reads in the biofilm from station 5 where the two strains PMC 1069 and 1070 were isolated.

### Polyphasic approaches and taxonomic affiliation

**Figure 4.**
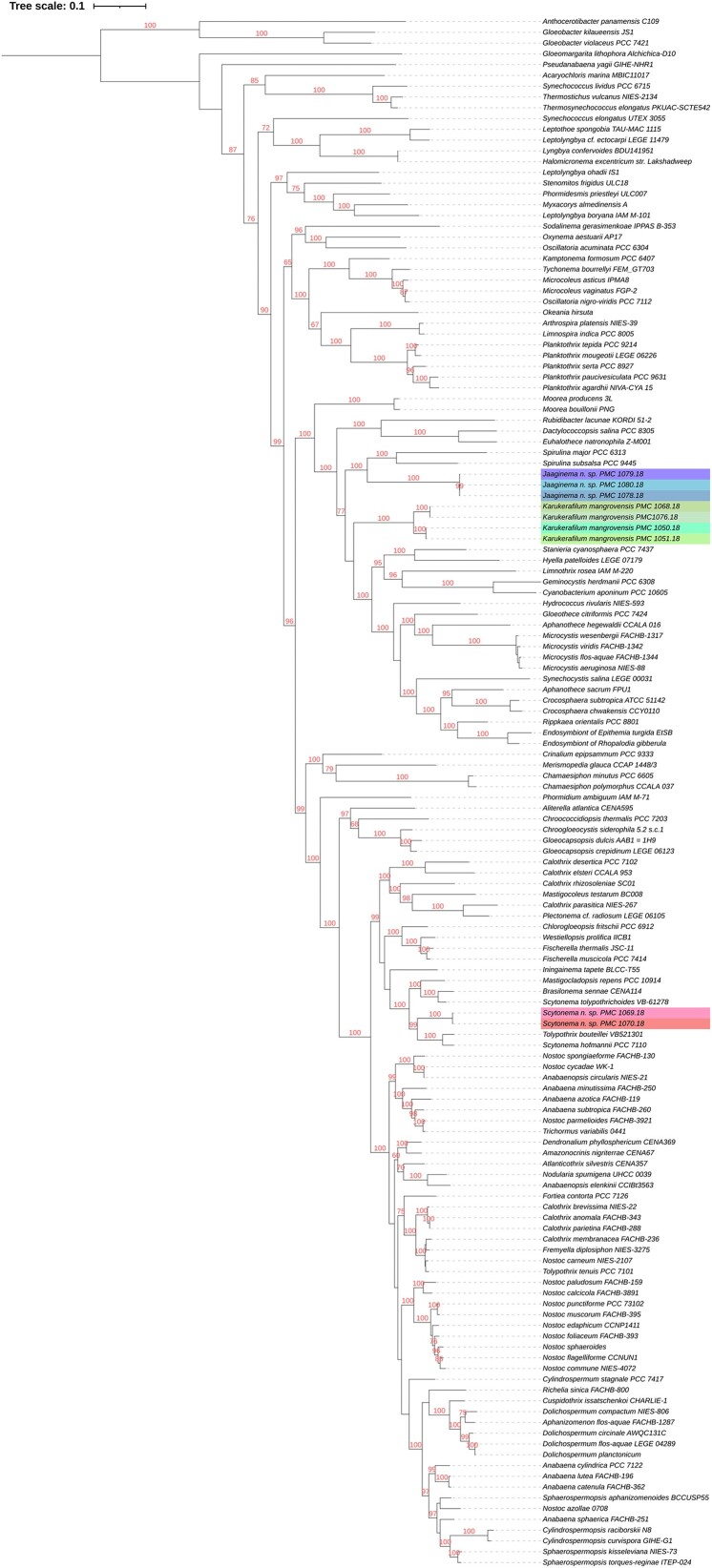
Phylogenomic tree based on the comparison of 30 concatenated genes from 144 available reference genomes (see text for methodology).

The strains PMC 1069 and 1070 grew as emerald-green isopolar filaments including barrel-shaped heterocytes (11.5 ± 0.7×18.6 ± 3.4 µm, Fig. 1A-B), displaying a ∼2 µm-thick sheath (Fig. 1C), and a false branching pattern, with no akinetes. Vegetative cells measured 13.2 ±1.0×14.3±1.0 µm (Table S1). Terminal cells appeared larger than long (10.6±0.8×8.8±0.8 µm, Fig. 1B-C; Table S1). Filaments were more or less constricted (Fig. 1D), constriction level being higher towards the terminal end. The two phylogenetic trees based on 16S rRNA sequences and the genomes (Fig. S2 and Fig. 4, respectively), clustered strains PMC 1069 and 1070 together. The phylogenomic tree based on concatenated sequences from 30 shared genes among 144 available reference genomes showed a bootstrap-supported sister clade to one clade containing *Tolypothrix bouteillei* and *Scytonema hofmanni* (Fig. 4) with average nucleotide identity (ANI) of 0.774 and 0.773, respectively. The polyphasic approaches with all the criteria used in this study are congruent with affiliation of PMC1069 and 1070 to the order Nostocales, family Scytonemataceae and genus *Scytonema* (Komarek et al. 2014).

Strains PMC 1078, 1079, and 1080 appeared as green tuft-like colonies of thin and uniseriate filaments, without sheaths (Fig. 2A-B). Vegetative cells are cylindrical and measured 2.9 ± 0.2 × 4.1 ± 0.8 µm (Table S1). Apical cells were elongated and hooked (Fig. 2A-B). Hormogonia were observed. A thin mucilage is visible around the filaments under the electron microscope, along with parietal localization of the thylakoids, and slight constriction at the cross walls between consecutive cells (Fig. 2C-D). Other components such as cyanophycin granules (nitrogen reserves) were observed within the cells (Fig. 2D). The strains PMC 1078, 1079 and 1080 appeared closely related together in the phylogenomic tree (Fig. 4, ANI >0.999). Their closest relative was a clade that contained two *Spirulina* (*S. salsa* and *S. major*). However, this clade was distant, ANI values were not high (0.720 to 0.723), and these strains did clearly not display the typical *Spirulina*-like coiled morphology (see Fig. 2). Morphological and ultrastructural features and the 16S rRNA-based phylogenetic clustering (Fig. S2) are congruent with the described genus *Jaaginema*, belonging to the Leptolyngbyales order (Anagnostidis and Komárek 1988, Mareš et al. 2019, Strunecký et al. 2023). Due to the lack of available sequences for *Jaaginema*, the phylogenomic tree alone is not conclusive regarding this affiliation (Fig. 4).

Strains PMC 1050, 1051, 1068 and 1076 displayed overall common features. They appeared as blue-green filaments, motile, with hormogonia (Fig. 3A). Cells were larger than long, or as large as long (5.84 ± 0.43 × 5.77 ± 1.28 µm for PMC 1050 and 4.72 ± 0.51 × 4.21 ± 0.97 µm for PMC 1068, Table S1). Cell content, and thylakoids with fascicular arrangement in the cytoplasm were visible (Fig. 3B-D). Morphological observations and 16S rRNA-based phylogenetic clustering (Fig. S2) are overall consistent with the Coleofasciculales (Komarek et al. 2014, Strunecký et al. 2023). The phylogenomic tree also clearly displayed a highly supported clade consisting of these four strains (bootstrap, BS: 100, Fig. 4). They clustered together, with no close relative, suggesting novelty and not allowing confident assignation to any of the existing genera (highest ANI <0.724). Their sister group consisted of representative of various genera belonging to several orders, and the grouping was not robust as evidenced by low bootstrap values (Fig. 4). Their previous affiliation to genus *Oscillatoria* (Duperron et al. 2020) is thus not supported based on the various criteria used in this study. Based on these results and the criteria required to characterize a new genus, that include 16S rRNA sequence similarity below 95% with existing genera combined with at least one autapomorphic character, habitat specificity and low average nucleotide identity (ANI), the classification of PMC 1050, 1051, 1068, and 1076 is proposed as a new genus and species. Thus, they were classified into the new genus *Karukerafilum* with the type species *K. mangrovensis*, described here as a benthic filamentous cyanobacterium associated to benthic mats from mangroves.

#### Formal description of the genus and species:

***Karukerafilum mangrovensis*** Halary, Duval, Marie, Bernard *et* Duperron, *gen. nov., sp. nov*.

The new genus *Karukerafilum* contains a single species (*K. mangrovensis*), thus a single description is provided for both genus and species.

**Description:** Thallus blue-green, thin, delicate, diffluent, forming thin mats. Trichomes solitary, cylindrical, more or less straight, flexuous, blue-green, motile, with hormogonia, slightly constricted at the cross walls, very slightly attenuated at the ends, Cells slightly wider than long (5.84 ± 0.43 × 5.77 ± 1.28 µm for PMC 1050 and 4.72 ± 0.51 × 4.21 ± 0.97 µm for PMC 1068). Cell content with radial or fasciculated thylakoid arrangement in the cytoplasm. Very thin-to-absent sheath, attached to the trichome.

**Diagnosis:** New genus differs from other genera by the substantial differences in the nucleotide sequence of the 16S rRNA-encoding gene and of 30 concatenated coding sequences.

**Type species (PMC 1050.18), here designated: *Karukerafilum mangrovensis*** Halary, Duval, Marie, Bernard *et* Duperron

**Holotype**: a cryopreserved and formaldehyde-fixed sample of the strain PMC 1050.18 was deposited at Paris Museum Collection (PMC), Paris, France. Corresponding reference 16S rRNA encoding sequence was deposited in GenBank under accession number MN823169 and an assembled MAG was deposited under accession SAMN36471718.

**Type locality:** Isolated from a dense benthic filamentous brown mat collected in the Manche-à-Eau lagoon, Guadeloupe (N 16.276°, W 61.555°).

**Etymology: ‘**Karukera’ is the name given to Guadeloupe by native Caribbean inhabitants before the arrival of Christopher Colombus and means 'island of beautiful waters’’; ‘filum’ stands for filament; ‘mangrovensis’ relates to the habitat of the strain.

### Inter-strain genomic heterogeneity

**Figure 5.**
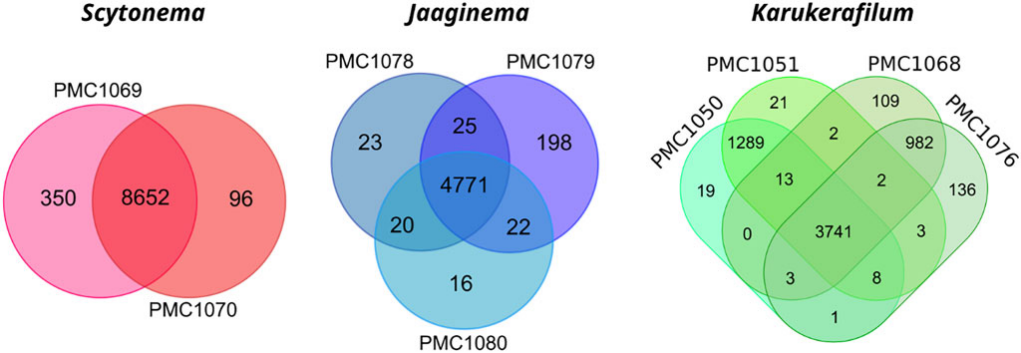
Venn diagrams showing the number of shared and unique ORFs for each of the three cyanobacterial lineages: *Scytonema* (left), *Jaaginema* (middle), *Karukerafilum mangrovensis* (right).

The *Scytonema*-related strains PMC 1069 and 1070 shared an ANI value above 0.999, and 7288 ORFs (Fig. 5, Table S2). They shared 8652 ORFs while 350 and 96 were unique to each strain, respectively. The *Jaaginema*-related strains PMC 1078, 1079, and 1080 had very few strain-specific ORFs (16 to 198 vs. 4 771 shared by all strains), and ANI values above 0.999. Interestingly, 20 to 25 ORFs were shared between two strains and absent from the third, showing a limited level of inter-strain differentiation. It must be noted that all three strains were isolated from the same biofilm sampled at station 7 (Table 1). The four *Karukerafilum mangrovensis* strains displayed a different picture. Overall, 3741 ORFs were shared among all four strains, and very few (19 to 136) were unique to a single strain. Interestingly, strains PMC 1050 and PMC 1051 shared additional 1289 ORFs unique to this clade, while PMC 1068 and PMC 1076 shared 982 (Fig. 5). Within each of these two subgroups, ANI values were above 0.999, while values between the two subgroups were 0.862, advocating for two distinct potential genotypes within a single new species (see below). Only a limited number of the ORFs that are differentially present among strains were annotated (33 out of 446 (7.4%) in *Scytonema*, 34 out of 304 (11.2%) in *Jaaginema*, and 161 out of 2591 (6.2%) in *K. mangrovensis*, Supplementary Table 3), the rest encoding for yet unknown functions.

### Genes involved in nitrogen and phosphorous metabolism

**Figure 6.**
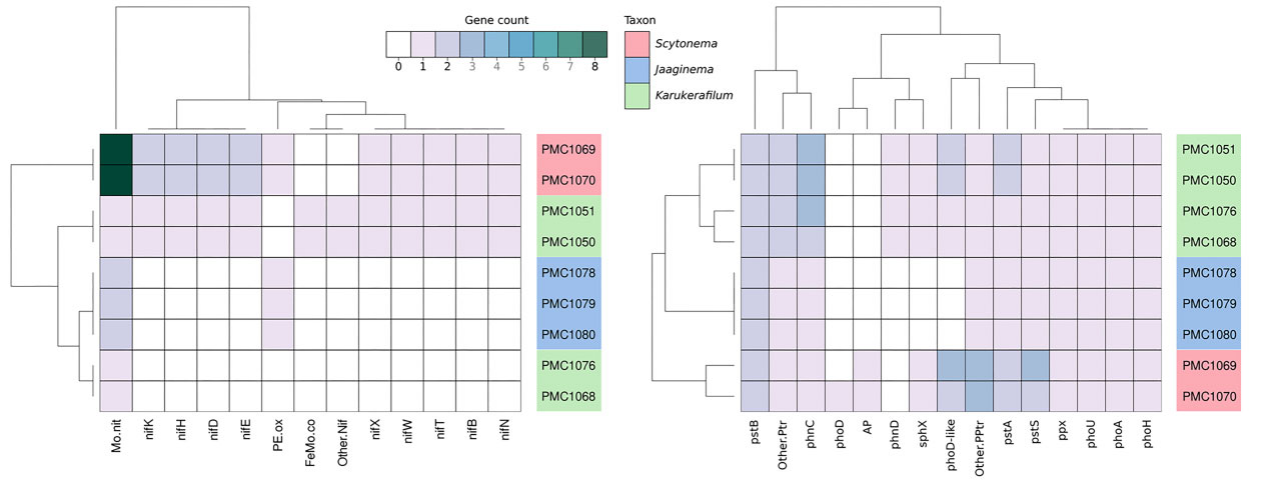
Occurrence and copy numbers of genes involved in nitrogen fixation and metabolism (left) and phosphorous metabolism (right).

*Scytonema*-assigned strains PMC 1069 and 1070 possessed nine *nif* genes (Fig. 6), including four as dual copies (*nifD, E, H, and K*) and Mo. Nit in 8 copies. *Jaaginema*-related strains PMC1078, 1079 and 1080 had none of these. On the other hand, gene-content heterogeneity was observed among the four *Karukerafilum mangrovensis* strains (Fig. 6, Table S3). Indeed, PMC 1050 and 1051, possessed ten *nif* genes as well as one gene involved in dinitrogenase iron-molybdenum cofactor (FeMo-co) synthesis. *A contrario*, none of these genes was found in strains PMC 1068 and 1076.

As for nitrogen-related genes, the two *Scytonema* strains displayed highly similar gene composition for genes involved in phosphorous metabolism, and the three *Jaaginema* strains displayed identical profiles (Fig. 6). Three genes differed among the two subgroups of *K. mangrovensis* strains, but only in terms of copy numbers (*phoD-like, phn*C*, pstA*, the former an alkaline phosphatase, the other two elements of the transport system).

### Secondary metabolites biosynthesis pathways

Biosynthesis pathways for secondary metabolites were searched for in all strains. *Scytonema*-affiliated genomes were those displaying the greatest number of putative pathways (Supplementary Table 4). Nonribosomal peptide synthetases (NRPS) were identified in *Scytonema* PMC 1069 and 1070. These included homologues with 100% similarity for the complete biosynthesis pathway of Anabaenopeptin NZ857/nostamide A found in *Nostoc punctiforme* PCC 73102 (three and one gene clusters, respectively). *Jaaginema*-assigned strains did not yield many matches with high similarity, the only complete and highly similar biosynthesis pathway was the one encoding for 1-heptadecene production. The *Karukerafilum mangrovensis* PMC1050 and PMC 1051 strains yielded a complete pathway for geosmin biosynthesis with 100% similarity, and two complete pathways for nostopeptolide A2 biosynthesis but with only 50% similarity (i.e. 50% of the genes had a significant BLAST hit to the genes). None of these was found in the two other *K. mangrovensis* strains PMC 1068 and 1076.

### Metabolomic profiling

**Figure 7.**
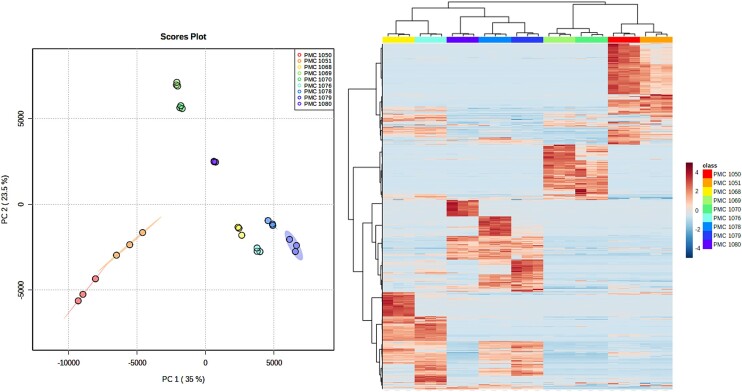
Metabolome profiling of the 9 strains. Left: Principal Component Analysis plot based on the analysis of 3836 analytes. Right: occurrence and intensity of MS-MS peaks corresponding to analytes (lines) in each strain (columns).

**Figure 8.**
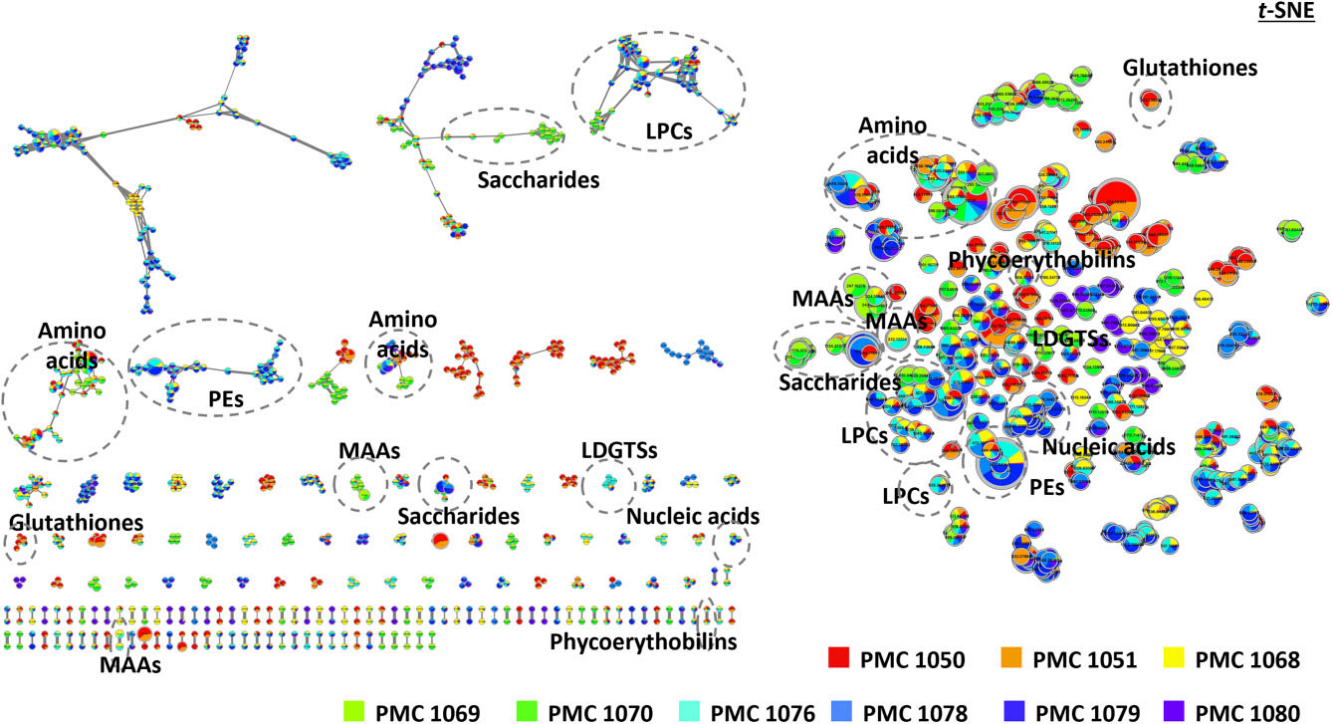
Network based on GNPS analysis displaying successfully annotated nodes, colors corresponding to the different strains in which nodes were identified.

Results from LC-MS positive and negative modes were assembled and compared among the nine strains. They yielded a total of 3836 metabolites, and strain clustering according to metabolome similarity was overall congruent with their respective affiliations, with closely related strains clustering together and sharing a higher proportion of their metabolites compared with other strains (Fig. 7). Although strains and cultures were non-axenic, we assume that most-to-all metabolites observed originated from cyanobacterial cells themselves, as most of the biomass in cultures corresponds to cyanobacteria. As for genomes and aforementioned gene contents, the four *Karukerafilum mangrovensis* strains were split in two groups, with PMC1050 and 1051 being distinct from PMC 1076 and 1068. Metabolite annotation revealed mostly primary metabolism, and very few secondary metabolites were successfully annotated (Fig. 8). For example, Anabaenopeptins, of which biosynthetic pathway genes were identified in strains PMC 1069 and 1070 were not detected despite the use of standards in our databases that should have permitted their identification, suggesting a lack of expression under culture conditions used. No production of other cyanotoxins was detected either under the culture conditions used in the laboratory.

## Discussion

### Novel strains representing genomes of poorly documented cyanobacterial taxa

Strains PMC 1069 and 1070 displayed the typical ultrastructure and morphology described for other *Scytonema*. Phylogenomics confirmed that they were related to *Tolypothrix bouteillei*, a filamentous nitrogen-fixing species isolated from building stones in India with a 11.5 Mb genome (Chandrababunaidu et al. 2015), and to *Scytonema hofmanni* PCC7110, also a nitrogen-fixer. As observed for these two close relatives, both strains also possess the *nif* genes and are thus able to fix nitrogen, a feature congruent with the occurrence of heterocytes in cultures.

The three strains PMC 1078, 1079 and 1080 can be classified as a single species based on phylogenetic, phylogenomic trees and the very high ANI similarity. They display the typical morphological features of the genus *Jaaginema*. Due to the lack of previously sequenced *Jaaginema* reference genome, their closest relatives are quite distant in the phylogenomic tree, consisting of *Spirulina salsa* and *S. major*. This distant relationship is likely due to a lack of available genomes, yet it was also observed in a recently published 16S rRNA-based phylogeny of several *Jaaginema* strains in which the same two *Spirulina* species were closest relatives of the various strains, yet with limited similarity (Brito et al. 2017). *Jaaginema* strains do not display heterocyts and do not possess the genes necessary for nitrogen fixation from N_2_ (Brito et al. 2017).

The strains PMC 1050, 1051, 1068 and 1076 were difficult to affiliate based on the 16S rRNA encoding gene, due to a limited number of closely related sequences, all from uncultured cyanobacteria (Duperron et al. 2020). The whole clade in this former tree was only distantly related to other Oscillatoriales, suggesting novelty. Whole genome sequencing confirms this novelty, with the four strains representing a new clade that is only distantly related to a group that contains genomes from numerous genera representing various cyanobacterial orders, with low ANI values, and does not support affiliation to any of the genomes available assigned to well-described (or known) genera. On the basis of 16S rRNA gene, strains were previously assigned to the Oscillatoriales, most likely family Oscillatoriaceae (Komarek et al. 2014, Duperron et al. 2020). Some features are compatible with features described in the genus *Oscillatoria* but this genus is reported to be polyphyletic and thus this genus-level classification is of limited relevance (e.g. (Hauerová et al. 2021)). Based on results from the present study, we propose that these four strains represent a new genus and new species with the type species *Karukerafilum mangrovensis* as a benthic filamentous mangroves cyanobacterium within the Order Coleofasciculales.

### Closely related strains display differences in gene content and potential capabilities

Analyzing two to three strains per clade revealed that closely related strains may harbor numerous unique ORFs despite very high ANI values. The four strains affiliated to *Karukerafilum mangrovensis* split into two sub-species-level subclades, each sharing its own additional set of ORFs beyond those shared by all four strains. This supports that the differentiation among strains involves differences in gene content, as recently described in large-scale comparisons of *Microcystis, Aphanizomenon* or *Limnospira* strains (Meyer et al. 2017, Pérez-Carrascal et al. 2019, Halary et al. 2023, Roussel et al. 2023). The ‘pan-genome’ concept has emerged as relevant to describe the metagenome of very closely related strains that cannot always be distinguished based on their 16S rRNA-encoding genes. The concept is particularly relevant to bloom-forming planktonic cyanobacteria in which distinct genotypes may bloom successively in what looks at first glance like a single-species bloom event (Beck et al. 2018). Based on our results, we suggest that its relevance should also be evaluated in the case of benthic biofilm-forming cyanobacteria, in particular because some of the non-shared genes are associated with important functions. Among the ORFs that are not common to all strains within each of the three clades, few were successfully annotated, suggesting that most of the heterogeneity is associated with unknown functions. However, some well-known functions did show inter-strain heterogeneity. An example is the occurrence of genes involved in nitrogen fixation in one of the two *K. mangrovensis* sub-species clades (strains PMC 1050 and 1051), but not the other (strains PMC 1076 and 1068). This suggests that only the former clade may have the ability to fix nitrogen, an important feature particularly in nitrogen limited habitats such as mangroves (Gontharet et al. 2017, Zilius et al. 2020). Various strains of nitrogen-fixing ”*Oscillatoria”*-like bacteria are reported, despite that these cyanobacteria do not develop heterocytes. Instead, certain cells usually localized in particular regions of the filament specialize into this metabolism (Bryceson and Fay 1981). Within-genus and even species differences in abilities to fix nitrogen were recently documented among strains of *Tolypothrix*, of which strain PCC 7712 can for example fix nitrogen while strain PCC 7601 does not (Bozan et al. 2022). Interestingly in this published report, both *Tolypothrix* strains possessed all necessary nitrogen fixation genes, the difference being hypothesized to result from inability of the latter to differentiate heterocytes.

Another type of heterogeneity between the two groups of *K. mangrovensis* strains is observed in the genes involved in phosphorous metabolism, with three genes displaying different copy numbers between the two groups. According to ANI values, these two sub-species clades belong to separate genotypes within *K. mangrovensis*, only one (PMC1050 and 1051) displaying genes for nitrogen fixation. Interestingly, strains form this subclade were isolated from the Manche-à-Eau lagoon, a seawater mangrove, while strains PMC 1068 and 1076 were isolated from periphytic biofilms at stations located in the Canal des Rotours, which water is slightly less salty (25 vs 35 g.l^−1^) in the upper layer of the water column where the biofilm samples were collected (Laverman et al. 2023). Mangrove habitats are known to be nitrogen-depleted (Fernandes et al. 2012), which may favor nitrogen-fixing strains. Nitrogen is reportedly available in the Manche-à-Eau lagoon, while NO_3_^−^ and NH_4_^+^ are available at low concentrations in the water column of the sites collected in the Canal des Rotours (Gontharet et al. 2017) respectively around 3 and 11 µM.

### Secondary metabolites potentially relevant to biofilm resistance to predation

Anabaenopeptin NZ857 biosynthesis gene clusters were identified in both strains of *Scytonema*. It was also identified in its relative *Tolypothrix bouteillei* VB521301. These hexapeptides inhibit phosphatases and proteases, which can induce toxicity against zooplankton (Lenz et al. 2019, Monteiro et al. 2021). They are reported from various cyanobacterial genera including *Anabaena, Nostoc, Microcystis, Planktothrix, Lyngbya*, and *Brasilonema*, the latter a close relative of *Scytonema* (Sanz et al. 2015). The variant NZ957 was specifically reported in *Synechococcus* sp. PCC 7502 and *Anabaena* sp. TAU NZ-3–1 (Monteiro et al. 2021). Induced toxicity to zooplankton could possibly play a major role in grazing limitation. However, anabaenopeptin was not detected in the metabolome of cultured strains, suggesting that its expression is not constitutive, and possibly occurs in conditions different from those used in our laboratory cultures.

Strains PMC 1050 and 1051 (*K. mangrovensis*) have the potential to produce geosmin, the volatile compound responsible for earthly odor. Recent work on *Caenorhabditis elegans* indicates that despite geosmin itself is not harmful to the worm, it is detected by this bacterivorous nematode and acts as an efficient repellent (Zaroubi et al. 2022). By possibly reducing grazing, geosmin production could be relevant to biofilm forming cyanobacteria. Zaroubi and co-workers suggested that geosmin could represent a chemical warning cue emphasizing the unpalatability of the producing bacteria, in their case a *Streptomyces*, in a way comparable to color patterns that induce learned avoidance responses in animals. Interestingly, strains PMC1050 and PMC 1051 are the two *K. mangrovensis* strains that have genes for nitrogen fixation, larger genomes, and these possibilities not shared with PMC 1076 and 1068, confirming that they belong to two different genotypes.

## Conclusion

Genome sequencing and polyphasic (*i.e*. phylogenetics, phylogenomics, ANI, morphological & ultrastructural characteristics, metabolites composition) analysis of nine selected strains abundant in benthic and epiphytic biofilms from Guadeloupe provides information regarding poorly explored cyanobacterial lineages, namely *Jaaginema, Scytonema* and a new genus *Karukerafilum* gen. nov. These three biofilm-forming clades display distinct abilities with regards to nitrogen fixation, phosphorous utilization as well as secondary metabolites biosynthesis potential, illustrating some possible successful strategies in the mangrove-to-freshwater transition zone. Besides their ability for primary production, an interesting feature is the genomic potential to produce toxins (*i.e*. anabaenopeptin) or repellent molecules (*i.e*. geosmin) that may prevent biofilms grazing, emphasizing biofilm-forming cyanobacteria as producers of potentially interesting bioactive molecules that warrant further exploration.

## Supplementary Material

xtad024_Supplemental_Files
