# Supplementary material for: Genomes of nine biofilm-forming filamentous strains of Cyanobacteria (genera Jaaginema, Scytonema, and Karukerafilum gen. nov.) isolated from mangrove habitats of Guadeloupe (Lesser Antilles)
Source: FEMS Microbes. 2023 Dec 14;5:xtad024. doi: 10.1093/femsmc/xtad024 (PMC10781437; doi:10.1093/femsmc/xtad024)
Supplement: xtad024_Supplemental_Files [file xtad024_supplemental_files.zip › Sup-Fig1-R1.pdf]

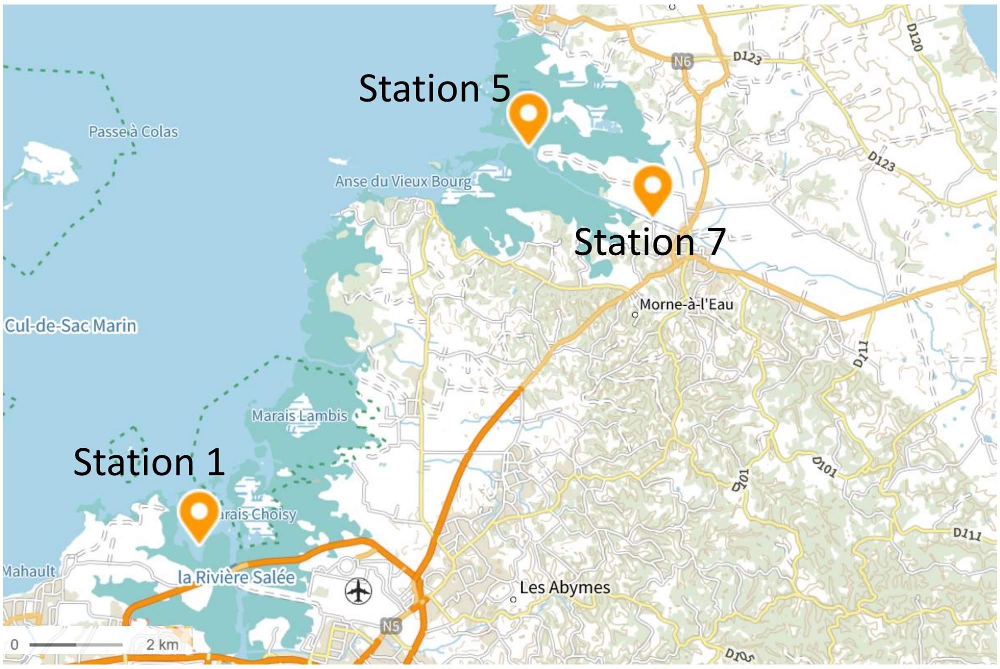

Supplementary figure 1: Map of the Grande Terre island, Guadeloupe, displaying the three sampled stations (manche-à-Eau, station 1; and stations 5 and 7 in the Canal des Rotours).
