# Supplementary material for: Genomes of nine biofilm-forming filamentous strains of Cyanobacteria (genera Jaaginema, Scytonema, and Karukerafilum gen. nov.) isolated from mangrove habitats of Guadeloupe (Lesser Antilles)
Source: FEMS Microbes. 2023 Dec 14;5:xtad024. doi: 10.1093/femsmc/xtad024 (PMC10781437; doi:10.1093/femsmc/xtad024)
Supplement: xtad024_Supplemental_Files [file xtad024_supplemental_files.zip › SupFig2-R1.pdf]

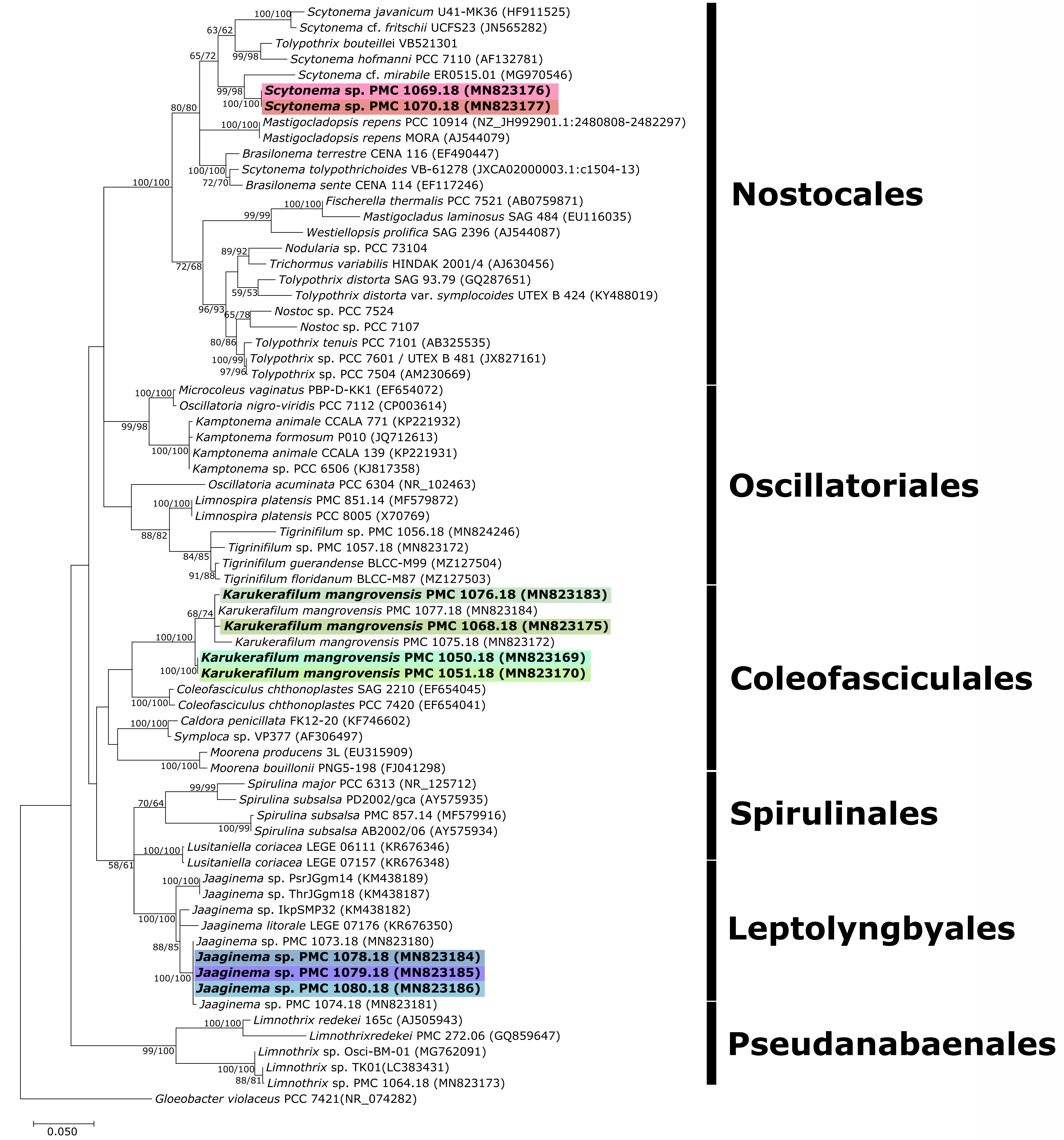

Scytonema javanicum U41-MK36 (HF911525)
Scytonema cf. fritschii UCFS23 (JN565282)
Tolypothrix bouteillei VB521301
Scytonema hofmanni PCC 7110 (AF132781)
Scytonema cf. mirabile ER0515.01 (MG970546)
Scytonema sp. PMC 1069.18 (MN823176)
Scytonema sp. PMC 1070.18 (MN823177)
Mastigocladopsis repens PCC 10914 (NZ_JH992901.1:2480808-2482297)
Mastigocladopsis repens MORA (AJ544079)
Brasilonema terrestre CENA 116 (EF490447)
Scytonema tolypothrichoides VB-61278 (JXCA02000003.1:c1504-13)
Brasilonema sente CENA 114 (EF117246)
Fischerella thermalis PCC 7521 (AB0759871)
Mastigocladus laminosus SAG 484 (EU116035)
Westiellopsis prolifica SAG 2396 (AJ544087)
Nodularia sp. PCC 73104
Trichormus variabilis HINDAK 2001/4 (AJ630456)
Tolypothrix distorta SAG 93.79 (GQ287651)
Tolypothrix distorta var. symplocoides UTEX B 424 (KY488019)
Nostoc sp. PCC 7524
Nostoc sp. PCC 7107
Tolypothrix tenuis PCC 7101 (AB325535)
Tolypothrix sp. PCC 7601 / UTEX B 481 (JX827161)
Tolypothrix sp. PCC 7504 (AM230669)
Microcoleus vaginatus PBP-D-KK1 (EF654072)
Oscillatoria nigro-viridis PCC 7112 (CP003614)
Kamptonema animale CCALA 771 (KP221932)
Kamptonema formosum P010 (JQ712613)
Kamptonema animale CCALA 139 (KP221931)
Kamptonema sp. PCC 6506 (KJ817358)
Oscillatoria acuminata PCC 6304 (NR_102463)
Limnospira platensis PMC 851.14 (MF579872)
Limnospira platensis PCC 8005 (X70769)
Tigrinifilum sp. PMC 1056.18 (MN824246)
Tigrinifilum sp. PMC 1057.18 (MN823172)
Tigrinifilum guerandense BLCC-M99 (MZ127504)
Tigrinifilum floridanum BLCC-M87 (MZ127503)
Karukerafilum mangrovensis PMC 1076.18 (MN823183)
Karukerafilum mangrovensis PMC 1077.18 (MN823184)
Karukerafilum mangrovensis PMC 1068.18 (MN823175)
Karukerafilum mangrovensis PMC 1075.18 (MN823172)
Karukerafilum mangrovensis PMC 1050.18 (MN823169)
Karukerafilum mangrovensis PMC 1051.18 (MN823170)
Coleofasciculus chthonoplastes SAG 2210 (EF654045)
Coleofasciculus chthonoplastes PCC 7420 (EF654041)
Caldora penicillata FK12-20 (KF746602)
Symploca sp. VP377 (AF306497)
Moorena producens 3L (EU315909)
Moorena bouillonii PNG5-198 (FJ041298)
Spirulina major PCC 6313 (NR_125712)
Spirulina subsalsa PD2002/gca (AY575935)
Spirulina subsalsa PMC 857.14 (MF579916)
Spirulina subsalsa AB2002/06 (AY575934)
Lusitaniella coriacea LEGE 06111 (KR676346)
Lusitaniella coriacea LEGE 07157 (KR676348)
Jaaginema sp. PsrJGgm14 (KM438189)
Jaaginema sp. ThrJGgm18 (KM438187)
Jaaginema sp. IkpSMP32 (KM438182)
Jaaginema litorale LEGE 07176 (KR676350)
Jaaginema sp. PMC 1073.18 (MN823180)
Jaaginema sp. PMC 1078.18 (MN823184)
Jaaginema sp. PMC 1079.18 (MN823185)
Jaaginema sp. PMC 1080.18 (MN823186)
Jaaginema sp. PMC 1074.18 (MN823181)
Limnothrix redekei 165c (AJ505943)
Limnothrixredekei PMC 272.06 (GQ859647)
Limnothrix sp. Osci-BM-01 (MG762091)
Limnothrix sp. TK01(LC383431)
Limnothrix sp. PMC 1064.18 (MN823173)
Gloeobacter violaceus PCC 7421(NR_074282)
0.050
Nostocales
Oscillatoriales
Coleofasciculales
Spirulinales
Leptolyngbyales
Pseudanabaenales
