# Supplementary material for: Genomes of nine biofilm-forming filamentous strains of Cyanobacteria (genera Jaaginema, Scytonema, and Karukerafilum gen. nov.) isolated from mangrove habitats of Guadeloupe (Lesser Antilles)
Source: FEMS Microbes. 2023 Dec 14;5:xtad024. doi: 10.1093/femsmc/xtad024 (PMC10781437; doi:10.1093/femsmc/xtad024)
Supplement: xtad024_Supplemental_Files [file xtad024_supplemental_files.zip › FEMSMC-2023-021 supplementary data - reviews and author response.docx]

**Reviews of manuscript FEMSMC-2023-021**

**Reviewer 1**

**Recommendation: Reject**

The paper focuses on the biology of the three cyanobacteria on the mangrove roots. Halar et al. sequenced genomes of 9 strains, reconstructed phylogenetic trees, compared metabolomes, and pangenomes of the strains, and performed 16S amplicon sequencing. Authors also attempt to propose a new species of cyanobacteria and they gathered a lot of information about their strains. Thus, it is unfortunate that the paper contains many inconsistencies and absent information, especially in the methods and results. Most importantly, the method of phylogenomic analysis is not mentioned at all, LM images have low magnification (is it impossible to see morphological features), and the taxonomic proposal is flawed. See details below.

Without extensive 16S phylogeny (it is mentioned, but I did not find it) and better photographs, I cannot evaluate if the taxonomic affiliations of two know species are correct as well as if the genus proposal could be made.

L 75, L200, Figs. Among the LM figures, I do not see any filament resembling Oscillatoria. Are you sure about that? Please, consult the paper https://www.preslia.cz/article/pdf?id=52 about the Oscillatoria type. Focus on the reproduction pattern and cell size.

L124. How did you isolate the strains?

What was the genome coverage?

Methods on phylogenetic reconstruction are missing. Please, explain all steps in detail.

L131and further. All tools must have settings explained or code included.

L143. Mentioned accessions contain reads not MAGs. I do not even think that MAGs are deposited in SRA.

A formal description has to be in the results. It is also incomplete. You have to provide a description of the type species for the genus. Karukerafilum cannot be a type of genus as it is now. If it is a type genus there would have to be a family described for this genus.

You do not state which code of nomenclature you use, but based on the provided information and contaminated cultures, you can only use botanical nomenclature (prokaryotic code accepts only axenic cultures). Please add the information about the code of nomenclature to the methods. Based on the botanical code, 16S sequence or genome sequence cannot be a type. You can have a sequence as a reference. Please distinguish between diagnosis and description:

https://onlinelibrary.wiley.com/doi/full/10.1002/tax.12200

L371-372. This statement does not belong to the etymology. I understand that you are saving it for the next paper, but at least one of those species needs to be added to the genus proposal. You can decide later if you have enough evidence for more than one species. Alternatively, you can change the paper conclusion to just say you probably found a new genus, but you will characterize it in the future.

L393-398. Yes, a lot of cyanobacterial species have nif genes and do not possess heterocytes (including Trichodesmium, which is Oscillatoriales), but it does not mean they fix nitrogen. Thus, your observation only suggests the difference in the gene composition, not the ‘ability to fix nitrogen’. Please, edit this part and specify your conclusion.

The images of the filaments in Figs 2 and 3 need to be under higher magnification. I cannot learn anything from them. Only Fig. 1 clearly represents Scytonema

Fig. 4. The supports are hard to read at the node. Also, the radial representation of the tree is extremely hard to read. I do not understand why did you root the tree to the Tychonema, Microcoleus clade and Gloeobacter lies among the other cyanobacteria. Please, check

https://www.sciencedirect.com/science/article/pii/S0960982221005777#undfig1

There must be some error.

the taxonomy seeks gaps between the species. In the discussion, you need to try to answer the question of how much difference is enough to describe a species based on the metabolome and pangenome data. It is well established that each strain has a different pangenome, which suggests a different metabolome. However, taxonomic applications have been discussed only recently because it took a long to have enough data to observe the patterns within and between the species diversity of the pangenomes, proteomes, metabolomes, and all the other omes in cyanobacteria.

Suppl. Fig. 1. This looks like a print screen from Google Maps. Please make sure that it could be reproduced like that in the paper considering the copyright. In any case, every map has to have scale. Moreover, station 7 seems to be inland and in an urban area. Can you specify the locality? Is there any marine water channel? I can see only land on the map.

**Reviewer 2: Alan Dobson, University College Cork**

**Recommendation: Minor Revision**

This paper describes, the morphological, metabolomic and genomic characterisation, all be it in quite a limited way on 9 cyanobacteria. The work is quite novel, particularly given the ecosysytem from which the bacteria have been isolated, the fact that the paper is reporting a new genus and the lack of genomic information in the current literature on isolates such as those being reported on. However the results section needs to be revised as it lacks clarity and the discussion section should focus more on the potential ecological significance of the metabolites and metabolic genes potentially encoded in the genomes.

The following need to be addressed.

Firstly there is a lack of clarity regarding why the 9 strains that were analysed were chosen for this study ? The rationale for choosing them is not clear.

The initial part of the results section is really confusing. It should start with the morphological data and then move to the genomic analysis. As written it is not clear. For example in line 196 they state Based on previous 16s rRNA sequence data etc (Figure 1) but figure 1 presents light microscopy images . In line 225 they state … low bootstrap values (Figure 2), but figure 2 presents more light microscopy images.

Phosphor metabolism is mentioned throughout the text, does this relate to Phosphate or Phosphorous?

The formal description of the genus lines 351 to 372 could potentially be presented as a supplementary table?

Line 374 should read .....and potential capabilities, as nothing has definitively been proven regarding production of anything. This should also be considered throughout the text. eg Line 407 … displaying capacities for nitrogen fixation. No activity has been demonstrated, but the potential capacity is there.

Line 430 states “ corresponding ASV representing half of the 16S rRNA reads” Where is this data presented in the text?

Line 435 states “Recent work on C. elegans indicates that geosmin itself is not harmful, but is detected by this bacterivorous nematode…” please clarify?

Line 379 needs to be reworded, it doesn’t make sense.

Other small editorial issues

Line 22 should read .. found abundantly..

Line 24 should read and searched for…

Line 32 for the genus

**Author's Response to Decision Letter for (FEMSMC-2023-021)**

Associate Editor's Comments to Authors:

Associate Editor

Comments to the Author:

Your paper has been seen by two expert reviewers. While they appreciate the aims of the paper in general, they also raise substantial criticisms that would need to be addressed thoroughly before we can consider this manuscript for publication in FEMS Microbes. In particular, please carefully consider the evidence required for your new taxonomic proposal.

We thank editor and reviewer for their time and their helpful comments, and have provided detailed answers below. A version of the manuscript with changes marked is available to make the review process easier.

We hope this revised version will be deemed suitable for publication,

With best regards

On behalf of authors,

Sébastien Duperron

Reviewers' Comments to Authors:

Reviewer: 1

Comments to the Author

The paper focuses on the biology of the three cyanobacteria on the mangrove roots. Halar et al. sequenced genomes of 9 strains, reconstructed phylogenetic trees, compared metabolomes, and pangenomes of the strains, and performed 16S amplicon sequencing. Authors also attempt to propose a new species of cyanobacteria and they gathered a lot of information about their strains. Thus, it is unfortunate that the paper contains many inconsistencies and absent information, especially in the methods and results. Most importantly, the method of phylogenomic analysis is not mentioned at all, LM images have low magnification (is it impossible to see morphological features), and the taxonomic proposal is flawed. See details below.

Without extensive 16S phylogeny (it is mentioned, but I did not find it) and better photographs, I cannot evaluate if the taxonomic affiliations of two know species are correct as well as if the genus proposal could be made.

L 75, L200, Figs. Among the LM figures, I do not see any filament resembling Oscillatoria. Are you sure about that? Please, consult the paper https://www.preslia.cz/article/pdf?id=52 about the Oscillatoria type. Focus on the reproduction pattern and cell size.

A 16S tree is now provided, after re-analysis of the already published 16S rRNA sequences versus a database of sequences from available and properly identified strains (supplementary figure 2).

Microscopy figures were revised (and associated material and methods added), in particular a higher magnification light microscopy image of Jaaginema was included in figure 2B; and figure 3 was modified to include a more detailed overview of a filament (3B), and the requested cross-section TEM micrograph (3C). A supplementary table was added with additional characteristics including measurements and comparison with related strains (supplementary table 1).

Altogether, these data allow to strengthen the affiliations.

L124. How did you isolate the strains?

Information was added “Another fraction was examined under a binocular and individual cyanobacterial morphotypes were isolated manually to plates containing solid Z8 medium (Rippka 1988) containing 0, 20, and 35 g.L-1 salt (Instant Ocean, Aquarium Systems, France). Cultured were stabilized and maintained in the Paris Museum Collection (PMC) (Duperron et al. 2020). “

What was the genome coverage?

Genome coverage data were added to table 1, ranging from 14.3 to 111.3x.

Methods on phylogenetic reconstruction are missing. Please, explain all steps in detail.

L131and further. All tools must have settings explained or code included.

A full detailed description is now added “Phylogenomic inference

All available reference genomes of Cyanobacteria with a complete assembly level in December 2021 were downloaded from NCBI (173 genomes in total), as well as a set of 3 genomes for an outgroup constitution, namely Anthocerotibacter panamensis C109, Gloeobacter kilaueensis JS1 and Gloeobacter violaceus PCC 7421. FetchMG was then used to retrieved single copy marker genes (MG) present in this dataset and our 9 cyanobacterial strain genomes (ref). In order to maximize the number of marker genes, genomes displaying a low number of MG were discarded. In total, 144 genomes sharing 30 MG altogether were kept for the phylogenomic analysis. First, a multiple alignment was achieved for each MG using MAFFT with local alignment iterative refinement option (ref). All alignments were then concatenated and refined using BMGE with default options (ref). Finally, the resulting alignment of length 6457 aa was used to build a phylogenomic tree using RaxML v8.2.12 (WAGGAMMA model, 100 bootstraps) (ref).“

L143. Mentioned accessions contain reads not MAGs. I do not even think that MAGs are deposited in SRA.

This has been corrected

A formal description has to be in the results. It is also incomplete. You have to provide a description of the type species for the genus. Karukerafilum cannot be a type of genus as it is now. If it is a type genus there would have to be a family described for this genus.

You do not state which code of nomenclature you use, but based on the provided information and contaminated cultures, you can only use botanical nomenclature (prokaryotic code accepts only axenic cultures). Please add the information about the code of nomenclature to the methods. Based on the botanical code, 16S sequence or genome sequence cannot be a type. You can have a sequence as a reference. Please distinguish between diagnosis and description:

https://onlinelibrary.wiley.com/doi/full/10.1002/tax.12200

As pointed by reviewer, we refer to the botanical code of nomenclature and use a polyphasic approach sensu Komarek. We reformulated the description accordingly in the results section, adding necessary data (see answer to first comment) as well as the description of the type species (see below).

L371-372. This statement does not belong to the etymology. I understand that you are saving it for the next paper, but at least one of those species needs to be added to the genus proposal. You can decide later if you have enough evidence for more than one species. Alternatively, you can change the paper conclusion to just say you probably found a new genus, but you will characterize it in the future.

We have added the description of the named species in the Karukerafilum genus: K. mangrovensis. Based on current evidence (ANI levels, gene contents, morphology, 16S rRNA), we propose a single species that includes two distinct genotypes

L393-398. Yes, a lot of cyanobacterial species have nif genes and do not possess heterocytes (including Trichodesmium, which is Oscillatoriales), but it does not mean they fix nitrogen. Thus, your observation only suggests the difference in the gene composition, not the ‘ability to fix nitrogen’. Please, edit this part and specify your conclusion.

We have tuned down the conclusion by indicating that “This suggests that only the former clade may have the ability to fix nitrogen…”

The images of the filaments in Figs 2 and 3 need to be under higher magnification. I cannot learn anything from them. Only Fig. 1 clearly represents Scytonema

Microscopy figures were revised, in particular a higher magnification light microscopy image of Jaaginema was included in figure 2B; and figure 3 was modified to include a more detailed overview of a filament (3B, Light microscopy), and the requested cross-section TEM micrograph (3C).

Fig. 4. The supports are hard to read at the node. Also, the radial representation of the tree is extremely hard to read. I do not understand why did you root the tree to the Tychonema, Microcoleus clade and Gloeobacter lies among the other cyanobacteria. Please, check

https://www.sciencedirect.com/science/article/pii/S0960982221005777#undfig1

There must be some error.

Thank you for pointing the wrong rooting of the tree, we had included the non-final version of the tree; we corrected this and used a classical rectangular visualization with the Gloeobacter as outgroup.

the taxonomy seeks gaps between the species. In the discussion, you need to try to answer the question of how much difference is enough to describe a species based on the metabolome and pangenome data. It is well established that each strain has a different pangenome, which suggests a different metabolome. However, taxonomic applications have been discussed only recently because it took a long to have enough data to observe the patterns within and between the species diversity of the pangenomes, proteomes, metabolomes, and all the other omes in cyanobacteria.

Thanks for this comment; we have modified the description to include a named species. As pointed by reviewer, in the light of current practice, we have limited evidence to support two species of Karukerafilum, and decided to propose a single species, which includes two slightly distinct genotypes.

Suppl. Fig. 1. This looks like a print screen from Google Maps. Please make sure that it could be reproduced like that in the paper considering the copyright. In any case, every map has to have scale. Moreover, station 7 seems to be inland and in an urban area. Can you specify the locality? Is there any marine water channel? I can see only land on the map.

Map was modified using Geoportail (free of copyright). Water channel is now visible, and scale is provided.

Reviewer: 2

Comments to the Author

This paper describes, the morphological, metabolomic and genomic characterisation, all be it in quite a limited way on 9 cyanobacteria. The work is quite novel, particularly given the ecosysytem from which the bacteria have been isolated, the fact that the paper is reporting a new genus and the lack of genomic information in the current literature on isolates such as those being reported on. However the results section needs to be revised as it lacks clarity and the discussion section should focus more on the potential ecological significance of the metabolites and metabolic genes potentially encoded in the genomes.

The following need to be addressed.

Firstly there is a lack of clarity regarding why the 9 strains that were analysed were chosen for this study ? The rationale for choosing them is not clear.

This was clarified “(strains)…were selected because they represent a significant fraction of the bacteria occurring in the biofilms from which they were isolated (Duperron et al. 2020), several strains are available, and because they represent lineages for which little genomic information is currently available in the literature. »

The initial part of the results section is really confusing. It should start with the morphological data and then move to the genomic analysis. As written it is not clear. For example in line 196 they state Based on previous 16s rRNA sequence data etc (Figure 1) but figure 1 presents light microscopy images. In line 225 they state … low bootstrap values (Figure 2), but figure 2 presents more light microscopy images.

We have reformulated the polyphasic approach and gathered all relevant data in a dedicated new section of the results. The 16S rRNA tree was added (figure S2), as well as new light and electron microscopy images that display the selected characters in a more appropriate way; as well as a table summarizing the morphological features (supplementary table 1) and finally have described a species to be the type species of genus Karukerafilum. Figure numbers were carefully checked.

Phosphor metabolism is mentioned throughout the text, does this relate to Phosphate or Phosphorous?

This refers to phosphorous, corrections were made.

The formal description of the genus lines 351 to 372 could potentially be presented as a supplementary table?

In accordance with the objective of the paper and comments from reviewer 1, we chose to let this in the main text but moved it to the results section.

Line 374 should read .....and potential capabilities, as nothing has definitively been proven regarding production of anything. This should also be considered throughout the text. eg

Title was changed to “Closely related strains display differences in gene content and potential capabilities“

Line 407 … displaying capacities for nitrogen fixation. No activity has been demonstrated, but the potential capacity is there.

Reworded “these two subclades belong to separate species, only one (PMC1050 and 1051) displaying genes for nitrogen fixation.”

Line 430 states “ corresponding ASV representing half of the 16S rRNA reads” Where is this data presented in the text?

This sentence was removed

Line 435 states “Recent work on C. elegans indicates that geosmin itself is not harmful, but is detected by this bacterivorous nematode…” please clarify?

Reformulated “Recent work on Caenorhabditis elegans indicates that despite geosmin itself is not harmful to the worm, it is detected by this bacterivorous nematode and acts as an efficient repellent”

Line 379 needs to be reworded, it doesn’t make sense.

reworded to “This supports that the differentiation among strains involves differences in gene content…”

Other small editorial issues

Line 22 should read .. found abundantly..

Done

Line 24 should read and searched for…

Done

Line 32 for the genus

Done

**Reviews of manuscript FEMSMC-2023-021.r1**

**Reviewer 1**

**Recommendation: Minor Revision**

I appreciate the authors' improvements to the paper. I have some additional minor comments.

L 20. “from”

L54. 1700 species does not seem to be correct. Check Algabase.org

L62 and onward. Genomics and taxonomy of cyanobacteria are discussed in https://www.sciencedirect.com/science/article/pii/S2211926423001613 and https://www.tandfonline.com/doi/full/10.1080/07352689.2020.1763541

L293. This is not diagnosis but description. Please consult https://onlinelibrary.wiley.com/doi/full/10.1002/tax.12200. Technically, you need the description for both new genus and species. But you can write below the following: “Karukerafilum mangrovensis Halary, Duval, Marie, Bernard et Duperron, gen. nov., sp. nov.” a sentence that the species description is the same as the genus description.

L302. The genus Karukerafilum has only a type species, not type genus. If you describe a new family you would propose the type genus.

L299-301. This sentence is diagnosis.

L303. Remove gen. nov., sp. nov.

L305-307. Sequences are not holotypes. Just mentions that you have ‘reference’ sequences for your holotype.

Metabolomic profiling. How did you treat contamination in your culture? Did you analyze only cyanobateria specific metabolites?

**Author's Response to Decision Letter for (FEMSMC-2023-021.r1)**

Dear editor,

Again, thanks for handling our submitted manuscript. Please find attached our details answers to reviewer 1 below:

Reviewer: 1

Comments to the Author

I appreciate the authors' improvements to the paper. I have some additional minor comments.

We thank reviewer for their very constructive comments that helped us improve the manuscript.

Q:L 20. “from”

A:Done

Q:L54. 1700 species does not seem to be correct. Check Algabase.org

A:Right, number is currently 5749, this was corrected, thanks

Q:L62 and onward. Genomics and taxonomy of cyanobacteria are discussed in https://www.sciencedirect.com/science/article/pii/S2211926423001613 and https://www.tandfonline.com/doi/full/10.1080/07352689.2020.1763541

A:Thanks, these two useful references are now cited (l. 70-72)

Q:L293. This is not diagnosis but description. Please consult https://onlinelibrary.wiley.com/doi/full/10.1002/tax.12200. Technically, you need the description for both new genus and species. But you can write below the following: “Karukerafilum mangrovensis Halary, Duval, Marie, Bernard et Duperron, gen. nov., sp. nov.” a sentence that the species description is the same as the genus description.

A: Diagnosis was modified to “description”; we added a sentence as suggested by reviewer “The new genus Karukerafilum contains a single species (K. mangrovensis), thus a single description is provided for both genus and species.“

Q: L302. The genus Karukerafilum has only a type species, not type genus. If you describe a new family you would propose the type genus.

A: Thanks, this was corrected to “Type species (PMC 1050.18), here designated: Karukerafilum mangrovensis…”

Q:L299-301. This sentence is diagnosis.

A: Thiis was corrected to “Diagnosis: New genus differs from other genera of the by the substantial differences in the nucleotide sequence of the 16S rRNA-encoding gene and of 30 concatenated coding sequences.“

Q:L303. Remove gen. nov., sp. nov.

A: Done

Q: L305-307. Sequences are not holotypes. Just mentions that you have ‘reference’ sequences for your holotype.

A: Sentence was reformulated “Corresponding reference 16S rRNA encoding sequence was deposited…” to avoid any ambiguity

Q: Metabolomic profiling. How did you treat contamination in your culture? Did you analyze only cyanobacteria specific metabolites?

A: In the absence of properly annotated massive databases of metabolites, only a limited number of metabolites can be annotated (these include those shown in figure 8), and it is rarely possible to assign individual metabolites to a given producing cell. The non-axenicity of our cultures may lead to occurrence of non-cyanobacterial metabolites, yet most of the biomass is cyanobacterial. This is now mentioned in the results (l. 370) “Although strains cultures were non-axenic, we assume that most-to-all metabolites observed originated from cyanobacterial cells themselves, as most of the biomass in cultures corresponds to cyanobacteria.”

Besides, previous work has shown that typical cyanobacteria-associated heterotrophic bacteria do usually not produce accessory/secondary metabolites (Demay et al 2020 Biomolecules; DOI:10.3390/biom11010028)
